# Liver diffusion-weighted MR imaging with L1-regularized iterative sensitivity encoding reconstruction based on single-shot echo-planar imaging: initial clinical experience

**DOI:** 10.1038/s41598-022-16324-x

**Published:** 2022-07-21

**Authors:** Maike Bode, Shuo Zhang, Mark N. Terwolbeck, Caroline Molavi Tabrizi, Paul Sprenger, Masami Yoneyama, Nils A. Kraemer, Christiane K. Kuhl, Alexandra Barabasch

**Affiliations:** 1 Department of Diagnostic and Interventional Radiology, University Hospital RWTH Aachen, Pauwelsstraße 30, 52074 Aachen, Germany; 2 Philips Healthcare, Hamburg, Germany; 3 Philips Healthcare, Tokyo, Japan

**Keywords:** Gastroenterology, Medical research

## Abstract

To investigate whether combining L1-regularized iterative sensitivity encoding (SENSE) reconstruction and single-shot echo planar imaging (EPI) is useful in hepatic DWI. Single-shot EPI-DWI with L1-regularized iterative SENSE reconstruction (L1-DWI) and conventional parallel imaging-based reconstruction (conv-DWI) in liver MRI were compared in volunteers and patients. For the patient cohort, 75 subjects (60 ± 13 years) with 349 focal liver lesions (FLL) were included. Patient groups A and B were used to reduce acquisition time or improve spatial resolution, respectively. Image parameters were rated on a 5-point scale. The number of FLLs was recorded; in case of discrepancy, the reason for non-detectability was analyzed. In volunteers, higher signal-to-noise ratio (24.4 ± 5.6 vs. 12.2 ± 2.3, *p* < 0.001 at b = 0; 19.3 ± 2.8 vs. 9.8 ± 1.6, *p* < 0.001 at b = 800) and lower standard deviation of the apparent diffusion coefficient-values (0.17 vs. 0.20 mm^2^/s, *p* < 0.05) were found on L1-DWI compared to conv-DWI. In patients, image ratings were similar for all parameters except for “conspicuity of FLLs” which was rated significantly lower on L1-DWI vs. conv-DWI (4.7 ± 0.6 vs. 4.2 ± 0.9, *p* < 0.05) in group A. In five patients, 11/349 FLLs were not detectable on L1-DWI, but on conv-DWI. L1-regularized iterative reconstruction of single-shot EPI DWI can accelerate image acquisition or improve spatial resolution. However, our finding that FLLs were non-detectable on L1-DWI warrants further research.

## Introduction

Numerous MRI techniques have been developed to accelerate acquisition time of MR imaging by skipping data in κ-space such as keyhole techniques, parallel imaging, and, more recently, compressed sensing (CS)^1–4^. First reported by Lustig et al. in 2007, CS uses the sparsity of image data and attempts to avoid undersampling artifacts by using incoherent undersampling with iterative reconstruction^5–7^. CS has been shown to yield comparable image quality compared to conventional imaging^8–10^. Most research projects focused on the use of CS to accelerate acquisition in dynamic contrast enhanced imaging of the liver and the breast, where fast image acquisition is of key importance^10–13^.

Diffusion weighted imaging (DWI) is a pulse sequence that could benefit from the principle of CS; not only for accelerated acquisition as the case with conventional parallel imaging, but also for improved signal-to-noise ratio (SNR) via denoising during iterative reconstruction^6^. Similarly, the faster acquisition can be invested to increase spatial resolution at a given acquisition time.

DWI plays an important role in the detection and characterization of focal liver lesions (FLLs)^14–16^. There is even evidence to suggest that DWI is more important for the detection of FLLs than standard structural liver MRI^17,18^. Recently wavelet-based denoising as used in the CS framework has been combined with parallel imaging that derives a L1-regularized iterative SENSE reconstruction showing effective in single-shot echo-planar imaging (EPI) based DWI noise reduction^19,20^.

Therefore, the aim of our study was to investigate CS-based reconstruction using L1-regularized iterative SENSE in EPI DWI (L1-DWI) in healthy volunteers and a cohort of patients undergoing liver MRI in direct comparison to conventional parallel imaging based reconstruction (conv-DWI), for both purposes, i.e. increased acquisition speed and increased spatial resolution.

## Material and methods

### Study design and inclusion criteria

This comparative analysis was conducted in accordance with the Declaration of Helsinki and was approved by the Ethics Committee at the RWTH Aachen Faculty of Medicine (EK 226/20). Written informed consent was waived.

#### Volunteer studies

For quantitative assessment of the signal-to-noise ratio (SNR) and apparent diffusion coefficient (ADC), L1-DWI was performed in healthy volunteers and compared to conv-DWI. Details are described below in MR Imaging and Image Analysis.

#### Patient studies

For investigation of its potential clinical utility, L1-DWI was applied in therapy-naïve patients, who received standard multi-parametric liver MRI for clinical indications from November 2019 through February 2020 for either search of liver metastases of a newly diagnosed malignancy or diagnosis or staging of primary liver tumors. All patients underwent a standardized multiparametric liver MRI protocol, and conv-DWI plus L1-DWI that were to be acquired in randomized order.

To investigate the potential utility of L1-DWI in terms of reduced acquisition time or improved spatial resolution, two different types of L1-DWI protocols were used:

During the first half of the study period (November through December 2019), patients underwent conv-DWI plus L1-DWI, where CS-based reconstruction was used to reduce acquisition time of conv-DWI (group A); during the second half of the study period (January through February 2020), CS-based reconstruction was invested to improve spatial resolution compared with conv-DWI (group B). Accordingly, in group A, L1-DWI was acquired with the same spatial resolution as conv-DWI, but faster; in group B, L1-DWI were acquired with similar acquisition time as conv-DWI but had a higher spatial resolution. In both patient groups, the same acceleration factor of 3 was applied as in healthy volunteer exams.

If a patient underwent more than one liver MRI study during the study period, only the first examination was included to avoid repetitive observations of the same patient to confound the results.

### MR imaging

All MR examinations were performed at a 1.5 Tesla system (Ingenia, Philips Heathcare, Best, The Netherlands). A torso phased array coil was used in all studies. The standard liver MRI protocol consisted of the following pulse sequences: axial breath-hold dual-echo T1 gradient echo in and opposed phase, axial T2-weighted turbo-spin echo (T2w TSE) sequence with and without spectral-selective fat suppression, coronal T2w TSE-sequence, a dynamic contrast enhanced series consisting of axial T1-weighted turbo field echo (Turbo i.e. gradient echo) sequence before and after contrast media injection (Gadobutrol or Gd-EOB-DTPB Dinatrium), followed by late phase enhanced-T1-weighted High Resolution Isotropic Volume Examination (eTHRIVE) using a fat-suppressed gradient-echo pulse sequence.

Liver DWI was performed before contrast media injection. For acquisition 2D multi-slice spin-echo single-shot EPI with respiratory belt triggering was used for both L1-DWI and conv-DWI. For L1-DWI acquisition, a moderate acceleration factor of 3 was applied based on empirical data from other clinical studies in anatomical imaging and volunteer results prior to the current study^3,9^. This provided relatively shorter EPI train length, echo time, and repetition time, compared to those in conv-DWI (acceleration factor 2), and resulted in a slightly reduced nominal scan time. For conv-DWI reconstruction, conventional SENSE was applied to the undersampled data. In addition to that, scan parameters of both sequences were matched as closely as possible, with shortest possible repetition times and same number of slices in the interleaved order in each case. For L1-DWI reconstruction, a regularized, iterative L1-norm minimization was applied assuring image sparsity in the wavelet domain and data consistency, as in typical CS reconstruction by taking the SENSE coil information into account^20,21,26,28^. Standard denoising was applied (Supplemental Digital Content 2). Image reconstruction was CPU-parallelized on the standard MR console computer (64 GB RAM, Intel Xeon E5–1620 CPU). Images were stored in the hospital database. Detailed MRI parameters of conv-DWI and L1-DWI for both volunteers and patient groups are listed in Table 1.

**Table 1:** MR imaging parameters of conv-DWI and L1-DWI based on single-shot EPI in this study.

MR parameters	conv-DWI	L1-DWI		
		Volunteers	Group A	Group B
Acceleration factor	2	3	3	3
Slice thickness (mm)	6	6	6	6
Matrix size	160 × 160	160 × 160	160 × 160	176 × 176
Acquired voxel size (mm³)	2.38 × 2.38 × 6	2.38 × 2.41 × 6	2.38 × 2.41 × 6	2.16 × 2.22 × 6
Reconstructed voxel size (mm³)	1.7 × 1.7 × 6	1.7 × 1.7 × 6	1.7 × 1.7 × 6	1.48 × 1.48 × 6
TR/TE (ms)	3981/86	3391/74	3391/74	3483/76
Flip angle	90	90	90	90
Number of signal acquisition (NSA)	4	4	4	4
Echo train length	69	45	45	49
EPI factor	69	45	45	49
Phase-encoding direction	AP	AP	AP	AP
Number of slices	31	31	31	31
b-values (s/mm^2^)	0/100/400/800	0/100/400/800	0/100/400/800	0/100/400/800
Nominal acquisition time (min:s)	3:31	3:00	3:00	3:24

*conv-DWI* Single-shot EPI-based DWI with parallel imaging reconstruction, *L1-DWI* Single-shot EPI-based DWI with L1-regularized iterative reconstruction.

### Image analysis

For image analysis, the images were saved in random order (L1-DWI vs. conv-DWI), anonymized for patient data (name, age, sex) and blinded for acquisition parameters (in particular including information on acquisition date, acquisition matrix, acquisition time).

#### Evaluation of volunteer studies

For signal-to-noise ratio (SNR) evaluation, additional noise maps were acquired for both L1-DWI and conv-DWI in volunteer measurements. The method was previously described^22–24^. In short, identical pulse sequence was performed without radio frequency excitation for the same imaging volume and separate noise-only images in each subject were obtained to account for influence from parallel imaging or CS reconstruction. As it prolonged the examination times, i.e., doubled corresponding L1-DWI or conv-DWI scan times, this evaluation was performed only in the volunteer studies. Regions of interest (ROIs) were carefully placed in the liver parenchyma of L1-DWI and conv-DWI with uniform signal without vessels or artifacts as well as in the corresponding noise map. Two ROIs were placed per volunteer. The mean values and standard deviations from each ROI were extracted to compute SNR and averaged for comparison. The same criteria of ROI selection also applied to quantification of the apparent diffusion coefficient (ADC).

#### Qualitative assessment of patient studies

For qualitative image analysis, two radiologists with 10 years (AB) and 4 years (MB) of experience in interpretation of abdominal MRI reviewed the CS- and conv-DWI data sets side-by-side, yet independently and blinded to the clinical information as described above. Image quality was visually scored according to a 5-point scale regarding the following features: (1) sharpness of the liver contours, (2) delineation of intrahepatic vessels, (3) signal homogeneity of the liver parenchyma and (4) conspicuity of FLLs, from 1 = poor to 5 = excellent; (5) image noise and (6) motion artefacts were evaluated using a 5-point scale ranging from 1 = severe to 5 = none.

#### Detection of focal liver lesions in patient studies

During a separate reading session done more than 16 weeks after the first session on image quality assessment, the two readers, again blinded to the type of DWI acquisition, reviewed the images in randomized order to compare the detection of FLLs. The number of FLLs for each DWI study of each patient was recorded.

When a FLL was missed on one of the pulse sequences (L1-DWI or conv-DWI), we investigated the respective cause: Whether the FLL was missed because of a reading failure, or whether, also in retrospect, i.e. with knowledge about the location of the FLL, the FLL was not visible; in this latter case, we recorded possible reasons for non-detectability such as local image degradation due to motion or pulsation artefacts.

In case of a discrepancy of the number of visible FLLs between L1-DWI and conv-DWI, we used all pulse sequences of the multiparametric hepatic MRI protocol, plus follow-up information of further MRI studies to clarify whether or not the DWI finding corresponded to a true FLL or to an artefact.

We also assessed the respective size and clinical relevance of the missed FLLs in terms of their implications on further patient management.

### Statistical analysis

Data were reported as mean and SD. Differences in continuous variables were evaluated with a paired *t*-test. For each image quality parameter, the mean and SD were calculated based on the ratings of the two readers and the resulting scores for L1-DWI and conv-DWI were compared by using a Wilcoxon rank sum test. The level of statistical significance was set at α = 0.05. The interrater variability was assessed by using weighted Cohens *Kappa* for each rated criterion at a 95% confidence interval. A coefficient value < 0.00 indicated poor agreement, 0.00–0.20 slight agreement, 0.21–0.4 fair agreement, 0.41–0.6 moderate agreement, 0.61–0.8 substantial agreement and 0.81–1.00 almost perfect agreement^25^. Statistical analyses were performed using IBM SPSS Statistics for Windows, version 25.0.

## Results

### Study cohort

**Figure 1:**
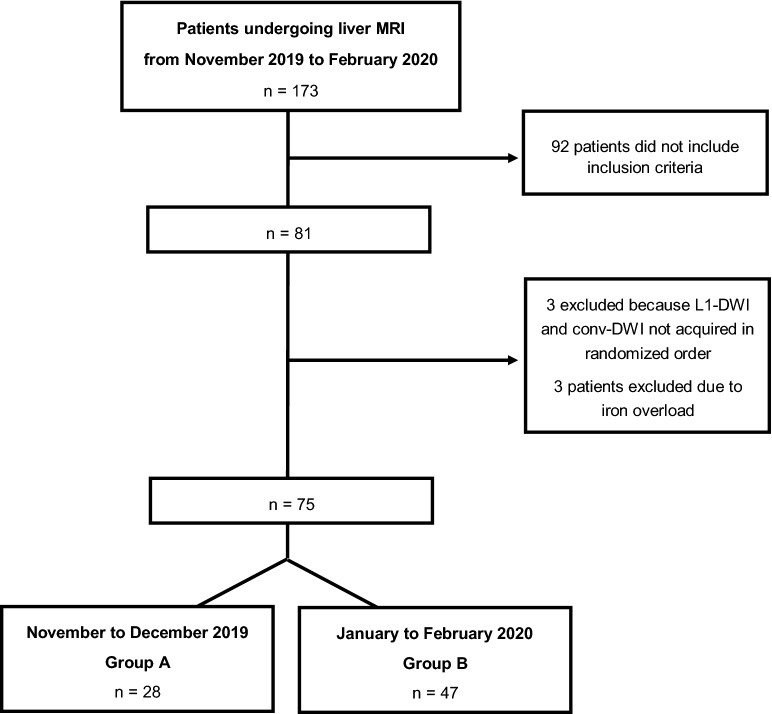
Flow diagram of the patient inclusion procedure.

Twelve healthy subjects (32 ± 7 years, 6 women) with different body sizes (1.57 to 1.93 m) and no known illness were included in the volunteer group. Figure 1 provides a CONSORT diagram of the final patient cohort. A total 81 consecutive patients (60 ± 13 years [mean ± SD], 34 women) undergoing liver MRI met the inclusion criteria for the clinical study. Three patients had not undergone conv-DWI or L1-DWI in randomized order. Another three patients underwent hepatocellular carcinoma screening because of known hemochromatosis; imaging studies of these patients had to be excluded because the iron overload yielded a non-diagnostic DWI data set regardless of the type of reconstruction.

Accordingly, the final patient cohort consisted of 75 patients. Patient demographics and details on their primary tumors are given in Table 2. Of the 75 patients, 28 were included during the first half of the study period (group A) and underwent L1-DWI to accelerate image acquisition compared to conv-DWI. Another 47 patients were included during the second half of study period (group B) and underwent L1-DWI with improved spatial resolution.

**Table 2:** Characteristics of the patients included in the study.

Characteristic		Patient data (n = 75)
Mean age (y)*	60 ± 13	
Sex	Men:women	41:34
**Staging in primary liver tumors**		**23**
	Hepatocellular carcinoma	13
	Cholangiocellular carcinoma	4
	Hemangiosarcoma	1
	Intraductal papillary neoplasms of the bile duct	1
	Focal nodular hyperplasia	2
	Hemangioma	1
	Adenoma	1
**Staging in primary tumors of other origin**		**52**
	Colorectal carcinoma	31
	Breast cancer	6
	Pancreatic cancer	5
	Melanoma	2
	Prostate cancer	1
	Cervical cancer	1
	Lymphoma	1
	Ovarian cancer	1
	Lung cancer	1
	Osteosarcoma	1
	Leiomyosarcoma	1
	Cancer of unknown primary	1
**Number of FLLs**		
	1–3	28
	4–6	11
	disseminated	17
**Accompanying disease**		
	Liver cirrhosis	8
	Ascites	7
	Pleural effusion	5

Of the 75 patients, 57 patients had at least one and up to 30 FLLs. In group A, 101 FLLs were observed in 20/28 patients. In group B, 248 FLLs were observed in 37/47 patients.

### Image analysis

#### Evaluation of volunteer studies

**Figure 2:**
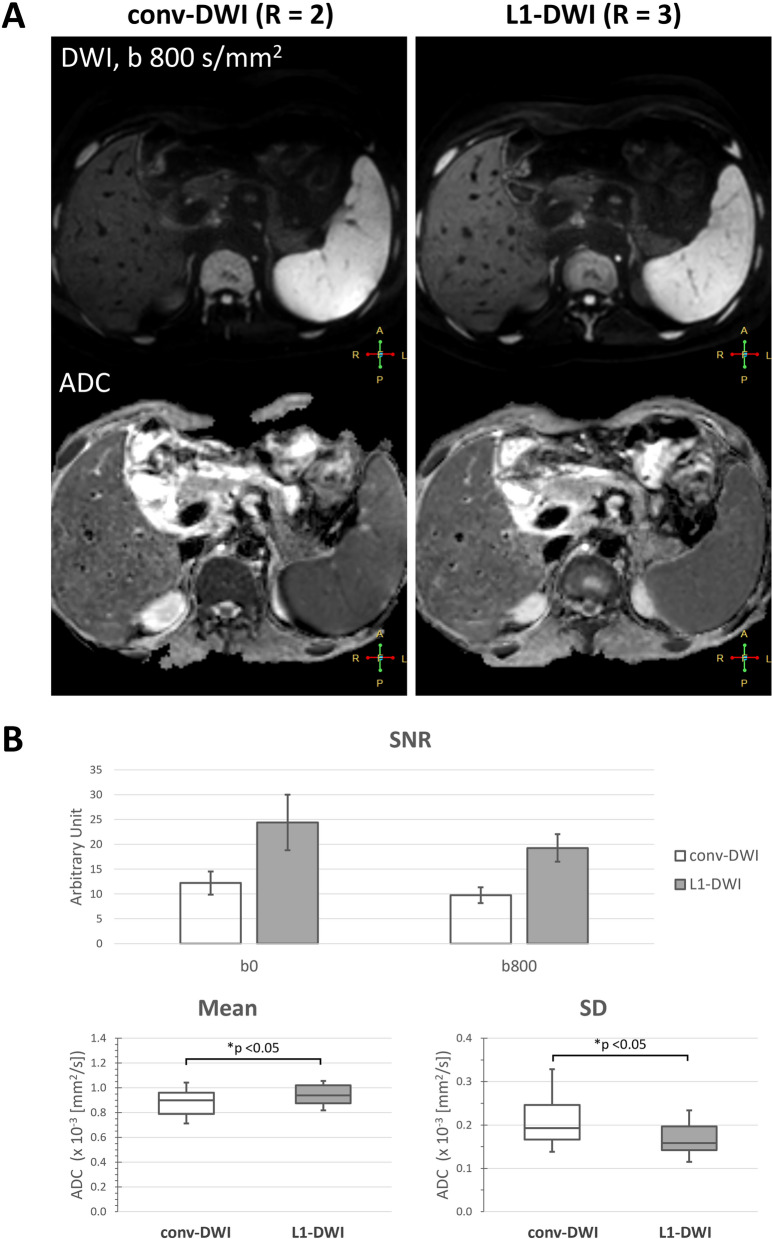
Comparison between liver conv-DWI (left) and L1-DWI (right) in healthy volunteers. conv-DWI = single-shot EPI-based DWI with parallel imaging reconstruction. L1-DWI = single-shot EPI-based DWI with L1-regularized iterative reconstruction. SD = standard deviation. (**A**) Images of a 35-year old volunteer of DWI at b = 800 s/mm^2^, and the resulting ADC image. (**B**) Higher SNR in DWI for both b = 0 and 800 s/mm^2^, as well as less variable ADC values were observed in L1-DWI in comparison to conv-DWI in 12 volunteers.

In general, reduced background noise and better structural delineation could be visualized in L1-DWI compared to conv-DWI, as shown in Fig. 2. In particular, SNR measurements showed almost a two-fold benefit in L1-DWI compared to conv-DWI for both b values of 0 s/mm^2^ (24.4 ± 5.6 vs. 12.2 ± 2.3, *p* < 0.001) and 800 s/mm^2^ (19.3 ± 2.8 vs. 9.8 ± 1.6, *p* < 0.001). Liver ADC values were deemed significantly different between L1-DWI and conv-DWI (0.94 ± 0.09 mm^2^/s vs. 0.88 ± 0.12 mm^2^/s, *p* = 0.006), whereas SD of ADC value in L1-DWI was found significantly lower than that in conv-DWI (0.20 vs. 0.17 mm^2^/s, *p* < 0.001).

#### Qualitative assessment of patient studies

Results are given in Table 3. In group A, most criteria used to analyze image quality yielded comparable ratings for L1-DWI vs. conv-DWI. However, conspicuity of FLLs was rated significantly lower for L1-DWI vs. conv-DWI (4.3 ± 0.9 vs. 4.7 ± 0.6, *p* = 0.006).

**Table 3:** Qualitative analysis results of the image quality from conv-DWI and L1-DWI in both patient groups. Mean scores of image quality parameters ± SD and range.

Image quality parameter		Group A			Group B		
		Conv-DWI	L1-DWI	*P*-value	Conv-DWI	L1-DWI	*P*-value
Sharpness of liver contours	Mean, SD [Range]	3.9 ± 0.8 [2–5]	4.0 ± 0.8 [2–5]	0.792	4.0 ± 0.7 [1–5]	4.0 ± 0.6 [1.5–5]	0.870
Intrahepatic vessel delineation	Mean, SD [Range]	3.8 ± 1.3 [1–5]	3.5 ± 1.2 [1–5]	0.106	3.6 ± 1.1 [1–5]	3.5 ± 1.1 [1.5–5]	0.079
Signal homogeneity of liver parenchyma	Mean, SD [Range]	3.9 ± 0.5 [3–4.5]	4.0 ± 0.5 [2.5–5]	0.157	3.8 ± 0.9 [1–5]	3.8 ± 0.9 [1–5]	1.000
Conspicuity of FLLs	Mean, SD [Range]	4.7 ± 0.6 [2.5–5]	4.3 ± 0.9 [2–5]	0.006*	4.5 ± 0.7 [2.5–5]	4.3 ± 0.9 [2–5]	0.056
Image noise	Mean, SD [Range]	3.4 ± 0.6 [2–4.5]	3.5 ± 0.7 [2–4.5]	0.617	3.3 ± 0.6 [1.5–4.5]	3.4 ± 0.7 [1.5–4.5]	0.425
Motion artifacts	Mean, SD [Range]	4.8 ± 0.5 [2.5–5]	4.8 ± 0.5 [2.5–5]	0.317	4.68 ± 0.7 [2–5]	4.7 ± 0.6 [2.5–5]	0.926

*conv-DWI* Single-shot EPI-based DWI with parallel imaging reconstruction, *L1-DWI* Single-shot EPI-based DWI with L1-regularized iterative reconstruction, *SD* Standard deviation. Significant *p*-values are highlighted with an asterisk.

*P*-values are from Wilcoxon signed-rank test.

**Figure 3:**
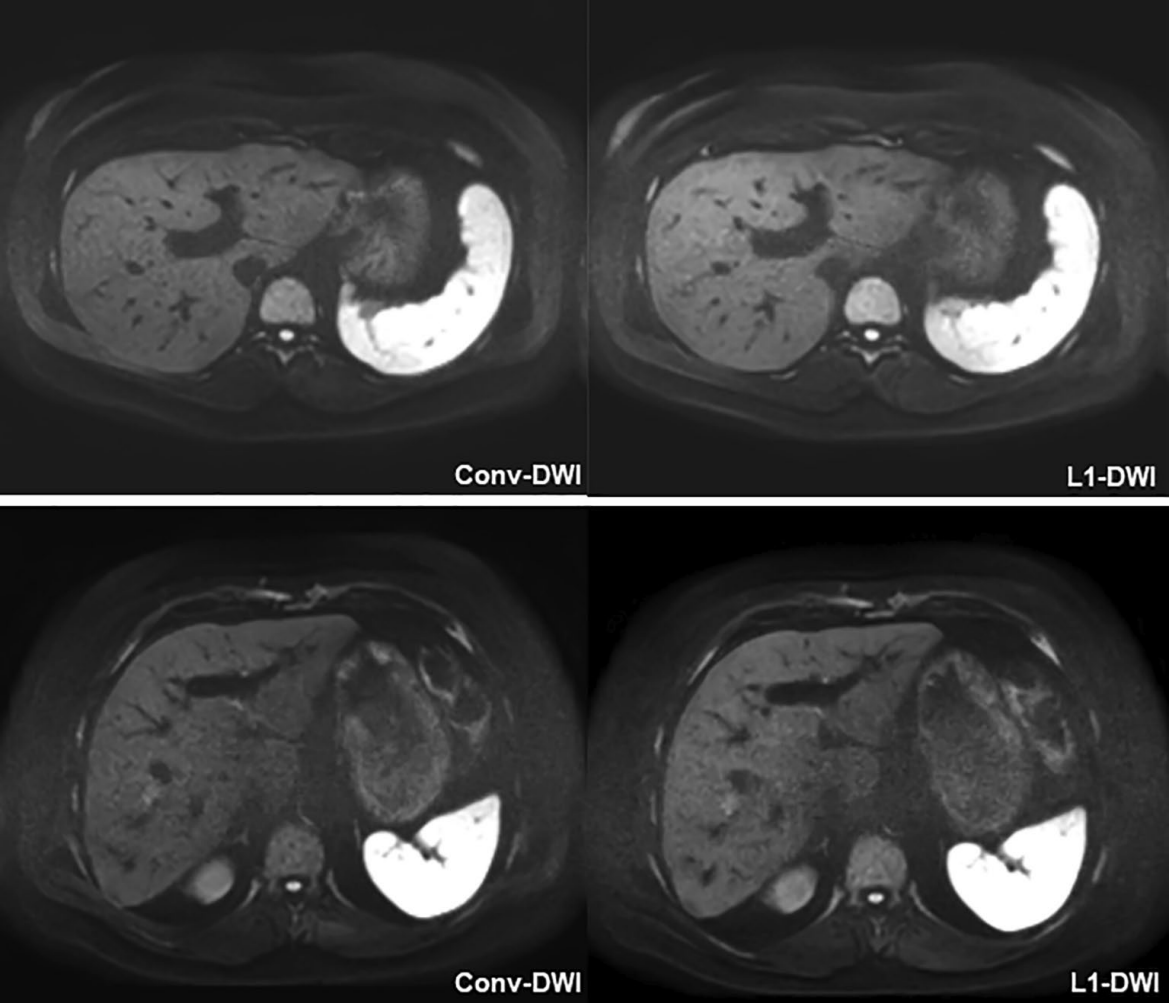
Images of conv-DWI and L1-DWI obtained with the L1-protocol in group A and group B. Representative images of conv-DWI and L1-DWI obtained with the CS-protocol in group A (upper row) and group B (lower row) yielding equivalent ratings for conv-DWI and L1-DWI.

In group B, image quality ratings did not differ significantly; although also here, a tendency for somewhat higher FLL conspicuity in conv-DWI was observed, this feature did not reach statistical significance. For representative images of group A and group B see Fig. 3.

The two readers showed slight to substantial agreement for the rated image quality parameters (see Table, Supplemental Digital Content 1, which provides data of interrater variabilities for both groups and each image criterion).

#### Detectability of focal liver lesions in patient studies

**Figure 4:**
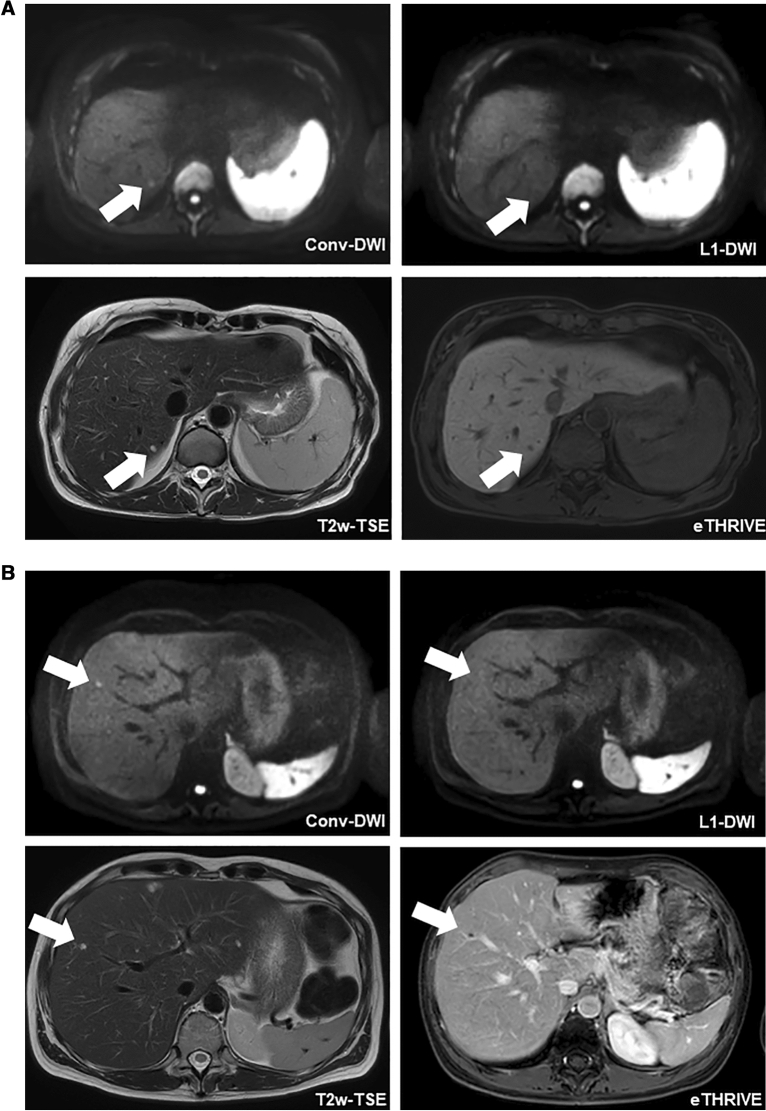
Patients of both groups with FLLs missed in L1-DWI. (**A**) 47-year-old female patient of group A who underwent a liver MRI for staging of colorectal carcinoma. In conv-DWI (upper row left), a FLL was detected in segment VII, that was not prospectively called on L1-DWI (upper row right). Taking all sequences from the routine MRI into account, e.g. T2-TSE (lower row left) and eTHRIVE (lower row right), this FLL corresponded to a cystic transformed metastasis. conv-DWI = single-shot EPI-based DWI with parallel imaging reconstruction. L1-DWI = single-shot EPI-based DWI with L1-regularized iterative reconstruction. T2w-TSE = T2-weighted turbo-spin echo. eTHRIVE = enhanced high-resolution fat-suppressed pulse sequence. (**B**) 46-year-old female patient of group B who underwent a liver MRI for staging of cervical carcinoma. In conv-DWI (upper row left), a FLL was detected in segment V, that was not prospectively called on L1-DWI (upper row right). Taking all sequences from the routine MRI into account, e.g. T2-TSE (lower row left) and eTHRIVE after injection of Gadobutrol (lower right right), this FLL corresponded to a thrombosed liver hemangioma. conv-DWI = single-shot EPI-based DWI with parallel imaging reconstruction. L1-DWI = single-shot EPI-based DWI with L1-regularized iterative reconstruction. T2w-TSE = T2-weighted turbo-spin echo. eTHRIVE = enhanced high-resolution fat-suppressed pulse sequence.

In 1 of the 28 patients of group A, a total of 2/101 FLLs (5 and 4 mm in diameter) were not detected on L1-DWI, but on the patient’s conv-DWI. Even in retrospect, these two FLLs were not visible on L1-DWI. An analysis of why these two FLLs were occult on L1-DWI showed that one FLL happened to be obscured by pulsation artefacts at the site of the FLL on L1-DWI. For the other missed FLL, no apparent reason could be identified (Fig. 4A).

None of the 28 patients in group A had FLLs detectable on L1-DWI that were not as well detectable on conv-DWI.

In 4 of the 47 patients of group B (6%), 9/248 FLLs (4%), between 3 and 7 mm in diameter, were not prospectively detected on L1-DWI, but prospectively called on the respective patient’s conv-DWI. Even in retrospect, these lesions were not visible on L1-DWI (Fig. 4B). An analysis of why the FLLs were occult on L1-DWI did not yield a definitive reason; in particular, there was no image degradation due to motion or pulsation artefacts at the site of the FLLs that would explain why FLLs were not visible.

In one of the 47 patients of group B, one FLL was not prospectively called on conv-DW, but prospectively detected on L1-DWI. This was explainable due to motion artefacts that degraded the area of the liver where the FLL was situated on conv-DWI, whereas there were no artefacts on L1-DWI.

In all cases of missed FLLs, a review of the medical records revealed that the detection of FLLs would not have altered patient management. Details of FLLs missed on L1-DWIL1-DWI or conv-DWI are given in Table 4.

**Table 4:** Patients with FLLs not detected on conv-DWI or L1-DWI.

	Number of FLLs		FLLs not seen		Location (liver segment)	Size (mm)	Type of FLL	Possible cause for non-detectability	Primary tumor
	Conv-DWI	L1-DWI	Conv-DWI	L1-DWI					
**Group A**									
Patient 1	6	4		-2	II/III VII	5 4	Metastases	Motion artefact None-conclusive	Colorectal carcinoma
**Group B**									
Patient 1	27	28	− 1		VI	4	Metastasis	Motion artefacts	Melanoma
Patient 2	17	16		− 1	IVa	5	Metastasis	None-conclusive	Colorectal carcinoma
Patient 3	26	20		− 6	II, V, VII, VIII	5–7	Metastases	None-conclusive	Pancreatic cancer
Patient 4	11	10		− 1	IVa	3	Liver cyst	None-conclusive	Colorectal carcinoma
Patient 5	3	2		− 1	IVb	4	Liver hemangioma	None-conclusive	Cervical cancer
Total of missed FLL			− 1	− 11					

*FLL* Focal liver lesion, *conv-DWI* Single-shot EPI-based DWI with parallel imaging reconstruction, *L1-DWI* Single-shot EPI-based DWI with L1-regularized iterative reconstruction.

## Discussion

In this intra-individual comparative study on image quality and diagnostic utility of diffusion weighted liver MRI with and without CS-based reconstruction, we found that IL1-DWI does allow a reduced image acquisition time and/or an improved spatial resolution, and yields about comparable image quality as conv-DWI. However, our results indicate that detectability of FLLs was impaired in L1-DWI, both at the subjective assessment of the conspicuity of visible FLLs, as well as at the quantitative analysis of FLLs seen or missed in the respective DWI protocols. Although FLLs missed on L1-DWI was only a small fraction of the overall number of lesions, it is particularly puzzling (or worrisome) to note that non-detectability of lesions in L1-DWI was not explainable by or attributable to e.g. artefact degradation, but was observed also in L1-DWI images that appeared to offer perfect image quality.

CS has been introduced to accelerate data sampling in MRI. Most studies on the clinical application of CS focused on its use to shorten acquisition time, and analyzed the resulting image quality. Most authors found that CS allows a reduction of image acquisition without loss of image quality—which is in good agreement with our findings. However, much less information is available on the diagnostic accuracy of such heavily accelerated pulse sequences. One study, by Nam et al. investigated the use of CS for dynamic contrast enhanced liver MRI including detection of FLLs in a multi-reader study and found a similar diagnostic accuracy of CS Dixon-gradient recall echo over standard non-CS Dixon-gradient recall echo. However, in that study, no head-to-head comparison of individual detected lesions was performed.

The observed SNR improvement in L1-DWI in volunteer scans most likely benefits from the iterative denoising process in the wavelet domain, in which the noise patterns are sparsely represented, as well as a shorted echo time due to higher acceleration factor used in L1-DWI. Lower standard deviations in ADC of L1-DWI have shown less variability and may indicate a higher precision and reproducibility in ADC assessment, which is in accordance to other studies^20,22,26^. Higher ADC values in L1-DWI compared to conv-DWI has also been reported previously in different anatomies including brain and prostate^20,26^. A possible cause may be a shorter repetition time ) used in L1-DWI^27^ or higher noise in conventional SENSE images using parallel imaging. However, in the current study a quantitative SNR improvement in volunteer scans was not reflected by qualitative scores in patient exams, where no improvement was shown for L1-DWI in image noise and artifacts rating. While there may be various factors contributing to this discrepancy, including local ROI selection vs. global visual inspection, body size and composition variabilities in the volunteer vs. patient cohorts, etc., detailed investigations are needed to study its impact in image quality and, particularly, delineation of normal appearing tissues and lesions^28^.

The positive impact of CS on image quality could be expected to improve detectability of FLLs, e.g. by reducing motion artefacts in protocols that exploit fast data acquisition with CS, or by shortening the echo train length and thus improving SNR in DWI with CS. On the other hand, with the undersampling that is employed for CS, it is conceivable that small lesions are “levelled out” and thus not displayed with the true signal intensity that they should have on DWI or structural MRI. Accordingly, there is a need for research that compares the detectability of such small lesions in heavily accelerated pulse sequences.

In our study, 2 and 4% of FLLs observed in 4 and 6% of patients of group A and B, respectively, were detected by conv-DWI, but were not visible on L1-DWI. The majority of patients in our cohort, including the five patients who had FLLs missed on L1-DWI, had multiple FLLs. Therefore, the missed FLLs would not have led to a change of patient management. However, it is possible that in future patients, e.g. with colorectal cancer in preparation of major liver surgery, detectability of even small additional metastases may do impact the treatment plan.

A further analysis of the type of FLLs missed on L1-DWI revealed that all missed FLLs were small, 7 mm or less in diameter, i.e. had a size similar to that of the section thickness of our DWI protocol. Small FLLs can always be missed on DWI in case there happens to be image degradation due to artefacts at the site of a lesion, or in case partial volume averaging leads to reduced contrast. In these latter situations, however, there should at least be a subtle signal intensity difference on a post-hoc analysis of the respective images—which was not the cases where FLLs were missed in L1-DWI in our study cohort. Also, a post-hoc analysis of image quality at the site of the missed FLLs revealed no artefact degradation as a possible explanation for such a misdetection.

One explanation of missed FLLs in L1-DWI in group B is because of their small size and reduced SNR in the image associated with higher spatial resolution. Moreover, the contrast to noise may be further decreased due to CS denoising that already occurred in every DWI measurement in the resource images. In this case, although the background tissue, i.e. liver parenchyma, appears smoother and less noisy via CS denoising, the visibility of small FLLs may become less apparent, which can no longer be retrieved by the sparsifying constraint in the CS-based reconstruction with L1-regularized SENSE including wavelet-based denoising, even after adding multiple measurements or acquisitions (see Figure, Supplemental Digital Content 2, which shows a numeric simulation of the effect of CS-based reconstruction with wavelet-based denoising in small lesion detection). In fact, although in line with most CS reconstruction techniques in clinical applications^9,27,28^, the same equidistant *k*-space acquisition pattern as in conventional single-shot EPI was used here for CS-based reconstruction, instead of a specifically tailored incoherent sampling pattern^3,6,7^. This might also limit the denoising performance. On the other hand, different imaging parameters between L1-DWI and conv-DWI such as echo times, and therefore scan time, may also contribute to lesion conspicuity, for example, due to induced image contrast change. Consequently, a systematic evaluation of the scan set ups in clinical settings is needed. The setting of the parameters should not only be chosen based on the image quality, but also based on the visualization of small FLLs.

Our study has several limitations. First, the study cohort was relatively small. In addition, the subgroups differed in the number of patients, which is due to the fact that the enrollment of patients was fixed at two months for each group in our study design. Second, the study cohort consisted of a heterogeneous patient population. On the other hand, this may also be considered advantageous because we assessed the feasibility of L1-DWI in various clinical settings. Third, most FLLs were not confirmed by pathology. Accordingly, although we did our best to provide a sound composite ground truth, it is conceivable that detected and non-detected small FLL did not correspond to a relevant pathologic condition. Finally, our study design did not include having fewer number of signal averages as another way to reduce the scan time^29^, in which case the minimum repetition time should be determined by T1 of the background tissue to reach thermal equilibrium^27,30^.

In conclusion, although L1-DWI can be used to reduce acquisition time or increase spatial resolution, and although measured SNR and stability of ADC values are improved, its image quality in clinical patients appears to be mostly comparable, while detection of FLLs is affected. Further investigations on optimal scan setups are needed before implementation of L1-DWI in clinical practice and should be done not only based on image quality but also visualization of small FLLs.

## Supplementary Information

Supplementary Information.

## Data Availability

All data generated or analyzed during this study are included in this published article [and its supplementary information files]. Data are however available from the corresponding author.

## Abbreviations

ADC: Apparent diffusion coefficient
conv-DWI: Single-shot EPI-based DWI with parallel imaging reconstruction
CS: Compressed sensing
L1- DWI: Single-shot EPI-based DWI with L1-regularized iterative reconstruction
DWI: Diffusion weighted imaging
EPI: Echo-planar imaging
eTHRIVE: Enhanced high-resolution fat-suppressed pulse sequence
FLL: Focal liver lesion
ROIs: Regions of interest
SENSE: Sensitivity encoding
SNR: Signal-to-noise ratio
T2w TSE: T2-weighted turbo-spin echo
