# Author Correction: Liver diffusion‑weighted MR imaging with L1‑regularized iterative sensitivity encoding reconstruction based on single‑shot echo‑planar imaging: initial clinical experience

**DOI:** 10.1038/s41598-022-22922-6

**Published:** 2022-11-04

**Authors:** Maike Bode, Shuo Zhang, Mark N. Terwolbeck, Caroline Molavi Tabrizi, Paul Sprenger, Masami Yoneyama, Nils A. Kraemer, Christiane K. Kuhl, Alexandra Barabasch

**Affiliations:** 1 Department of Diagnostic and Interventional Radiology, University Hospital RWTH Aachen, Pauwelsstraße 30, 52074 Aachen, Germany; 2 Philips Healthcare, Hamburg, Germany; 3 Philips Healthcare, Tokyo, Japan

Correction to: *Scientific Reports* 10.1038/s41598-022-16324-x, published online 21 July 2022

Paul Sprenger was omitted from the author list in the original version of this Article.

The Author Contributions section now reads:

Conceptualization, A.B. and C.K.K.; methodology, M.B., S.Z. and N.A.K.; investigation, M.B., M.N.T. and C.M.T.; data curation, M.B., P.S.; resources, C.K.K.; writing—original draft preparation, M.B.; writing—review and editing, all authors; supervision, A.B. and C.K.K.; All authors have read and agreed to the published version of the manuscript.

The original Article has been corrected.

