# Supplementary material for: Liver diffusion-weighted MR imaging with L1-regularized iterative sensitivity encoding reconstruction based on single-shot echo-planar imaging: initial clinical experience
Source: Sci Rep. 2022 Jul 21;12:12468. doi: 10.1038/s41598-022-16324-x (PMC9304342; doi:10.1038/s41598-022-16324-x)
Supplement: Supplementary file 1 — Supplementary Information. [file 41598_2022_16324_MOESM1_ESM.docx]

**Supplemental material 1.** Interobserver agreement of image quality parameters of group A and group B calculated by using a weighted Cohan’s kappa test.

conv-DWI = single-shot EPI-based DWI with parallel imaging reconstruction. L1-DWI = single-shot EPI-based DWI with L1-regularized iterative reconstruction.

| Image Quality Parameter | Group A |  | Group B |  |
| --- | --- | --- | --- | --- |
|  | Conv-DWI | L1-DWI | Conv-DWI | L1-DWI |
| Sharpness of liver contours  [95% CI] | 0.21  [-0.06, 0.48] | 0.38  [0.21, 0.75] | 0.34  [0.07, 0.61] | 0.34  [0.15, 0.54] |
| Intrahepatic vessel delineation  [95% CI] | 0.56  [0.32, 0.81] | 0.48  [0.21, 0.75] | 0.34  [0.17, 0.52] | 0.53  [0.37, 0.69] |
| Signal homogeneity of liver parenchyma  [95% CI] | 0.30  [0.01, 0.60] | 0.40  [0.02, 0.78] | 0.45  [0.24, 0.67] | 0.51  [0.31, 0.72] |
| Conspicuity of FLLs  [95% CI] | 0.20  [-0.12, 0.52] | 0.33  [0.07, 0.59] | 0.29  [0.12, 0.46] | 0.63  [0.47, 0.80] |
| Image noise  [95% CI] | 0.43  [0.14, 0.71] | 0.52  [0.27, 0.77] | 0.25  [0.04, 0.46] | 0.29  [0.09, 0.49] |
| Motion artifacts  [95% CI] | 0.35  [0.02, 0.71] | 0.45  [0.11-0.80] | 0.53  [0.21, 0.85] | 0.42  [0.13, 0.71] |

**Supplemental material 2.** Numeric simulation of the effect of compressed sensing (CS) reconstruction in small lesion detection.
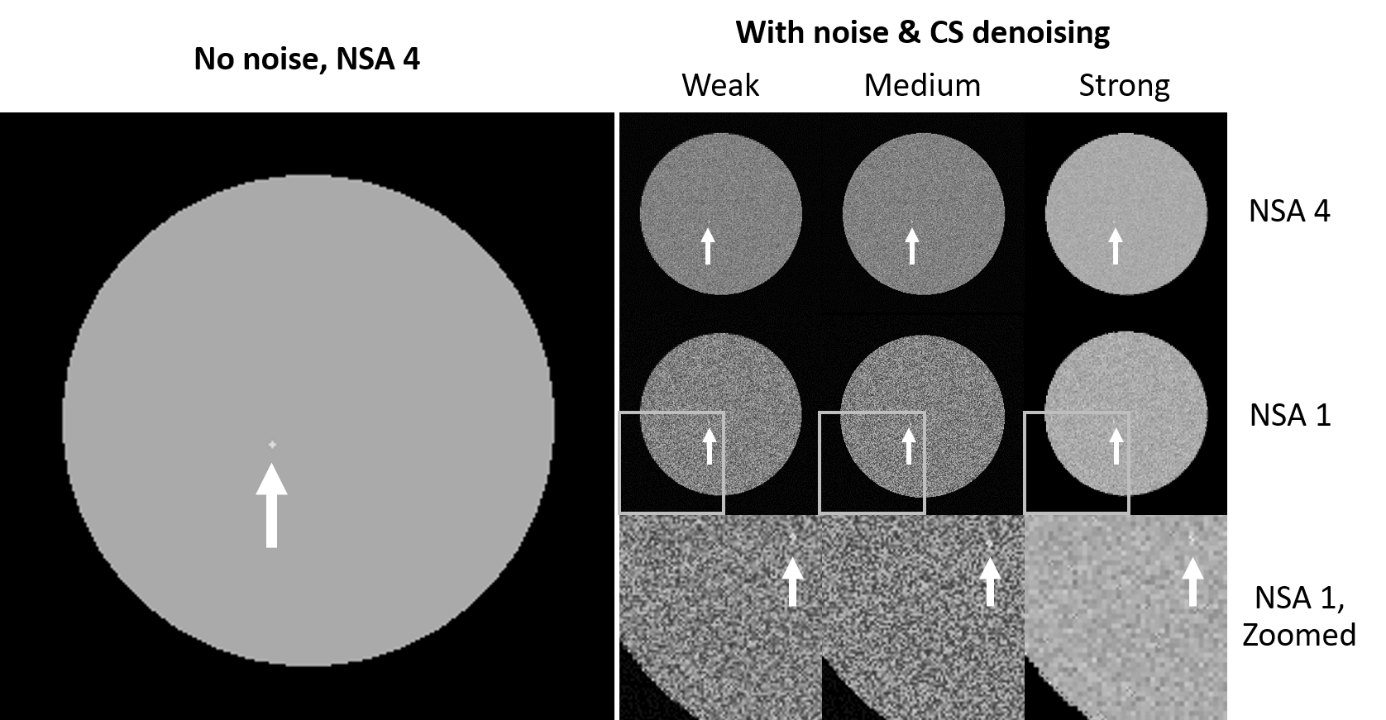


A phantom (matrix 256 x 256) with a circular uniform background and a focal spot (5 pixels) in the lower left quadrant was used. Same CS acceleration factor of 3 was used in all cases. Image without noise and with 4 averages (NSA 4) was shown as reference (*left*); images by adding noise and different CS denoising levels (weak, medium, strong) were compared (*right*). Averaged images (NSA 4) were displayed at the top, while single-average (NSA 1) images and a zoomed view at the focal spot quadrant were demonstrated at the middle and bottom rows. With stronger denoising level, although the background was becoming smoother and less noisy, the visibility of the focal spot became less apparent. This effect was obvious here because the spot, i.e. lesion size was small and its contrast to noise was also reduced at stronger denoising level, in which case the sparsifying constraint in CS reconstruction can no longer retrieve it.
